# Metabolic syndrome, serum uric acid and renal risk in patients with T2D

**DOI:** 10.1371/journal.pone.0176058

**Published:** 2017-04-19

**Authors:** Francesca Viazzi, Pamela Piscitelli, Carlo Giorda, Antonio Ceriello, Stefano Genovese, Giuseppina Russo, Pietro Guida, Paola Fioretto, Salvatore De Cosmo, Roberto Pontremoli, the AMD-Annals Study Group

**Affiliations:** 1 Università degli Studi and IRCCS Azienda Ospedaliera Universitaria San Martino-IST, Genova, Italy; 2 Department of Medical Sciences, Scientific Institute “Casa Sollievo della Sofferenza”, San Giovanni Rotondo (FG), Italy; 3 Diabetes and Metabolism Unit, ASL Turin 5, Chieri (TO), Italy; 4 Institut d'Investigacions Biomèdiques August Pii Sunyer (IDIBAPS) and Centro de Investigación Biomédicaen Red de Diabetes y Enfermedades Metabólicas Asociadas (CIBERDEM), Barcelona, Spain; 5 Department of Cardiovascular and Metabolic Diseases, IRCCS Gruppo Multimedica, Sesto San Giovanni, Milano, Italy; 6 Department of Clinical and Experimental Medicine, University of Messina, Messina, Italy; 7 Associazione Medici Diabetologi, Rome, Italy; 8 Department of Medicine, University of Padua, Padova, Italy; The University of Tokyo, JAPAN

## Abstract

**Background and aims:**

Metabolic Syndrome (Mets) and increased serum uric acid (SUA), are well known renal risk predictors and often coexist in patients with type 2 diabetes (T2D). Whether they independently contribute to the onset of CKD is at present unclear.

**Methods and results:**

Within the AMD Annals database we identified patients with T2D and normal renal function and urine albumin excretion at baseline and regular follow-up visits during a 4-year period. Blood pressure, BMI, HDL, triglycerides, and SUA were available in 14,267 patients. The association between Mets and/or hyperuricemia (HU, top fifth gender specific quintile) and the occurrence of renal outcomes were evaluated.

**Results:**

At baseline 59% of patients (n = 8,408) showed Mets and 18% (n = 2,584) HU. Over the 4-year follow-up, 14% (n = 1,990) developed low eGFR (i.e. below 60 mL/min/1.73 m^2^), and 26% (n = 3,740) albuminuria. After adjustment for confounders, BP≥130/85, low HDL, triglycerides ≥150 and HU were independently related to the development of low eGFR (1.57, P<0.001; 1.13, P = 0.056; 1.18, P = 0.008; 1.26, P = 0.001) and of albuminuria (1.35, P<0.001; 1.18, P = 0.001; 1.15, P = 0.002; 1.24, P = 0.001), respectively.

The incidence of low eGFR was higher in patients with HU independent of the presence or absence of Mets (21%, OR 1.30, p = 0.009 and 20%, 1.57, p<0.000 respectively), while albuminuria occurred more frequently in those with Mets and HU (32%, OR 1.25, p = 0.005) as compared to the reference group.

**Conclusions:**

HU and Mets are independent predictors of CKD and its individual components in patients with T2D.

## Introduction

Chronic kidney disease (CKD) is becoming a major global public health concern and its prevalence and incidence are steadily increasing mostly because of the rising burden of type 2 diabetes (T2D) and obesity worldwide [1].

Metabolic Syndrome (Mets) is widely prevalent among patients with CKD and has been reported to play a role in the progression of renal damage [2, 3] and development of end stage renal disease (ESRD) [4]. Moreover, Mets is increasingly recognized as an important predictor for de novo incidence of CKD, largely based on studies conducted among non diabetic populations from different ethnic groups [5–8]. So far, only one prospective study [9] conducted among Chinese patients with T2D investigated the relationship between Mets and the incidence of eGFR <60 mL/min/1.73 m^2^. A few studies examined the associations of Mets and the risk for albuminuria or proteinuria [10–14] but none among patients with diabetes.

Mets is also associated with hyperuricemia [15], which has been shown by some although not all studies to independently predict the onset and progression of CKD in several clinical settings, including T2D [16, 17, 18]. The associations between each individual component of Mets and outcomes have been reported to vary in the literature but are not thought to be sufficient to account for the increased hazard of CKD usually associated with Mets. It has been proposed that the components of Mets may foster the progression of renal damage mainly through the coexistence of several underlying pathological mechanisms such as increased oxidative stress, chronic inflammation, increased fibrogenic activity, and endothelial dysfunction [19]. Nevertheless, a causal relationship has not been proven and more studies are needed to precisely elucidate the mechanisms linking Mets to the development of renal damage.

The primary focus of this analysis was to examine the independent and joint associations of Mets, its components and increased SUA levels with risk of incident CKD and its components during a four year follow-up within a large registry of adults with T2D from across Italy.

## 1. Methods

### 1.1. Study setting, patients and data sources

The study database is representative of a large cohort of patients with T2DM followed-up at 131 diabetes centers in Italy and participating in the Italian Association of Clinical Diabetologists (Associazione Medici Diabetologi, AMD) initiative. The analysis was performed using electronic medical records collected between 1^st^ January, 2004 and 30^th^ June, 2008. For the purpose of the analysis, we considered only patients ≥18 years old and with a follow-up evaluation within 48±6 months complete for data about body mass index (BMI), blood pressure (BP) values, HDL-cholesterol, triglycerides, SUA, estimated GFR (eGFR) and urinary albumin excretion and information on treatment. Of 31,480 patients identified, we excluded 17,213 patients for the reasons detailed in S1 Fig. Fourteen-thousands-two-hundred-sixty-seven patients with T2D and from 69 clinics constitute the study population. The centers involved in the study were homogeneously distributed throughout the country.

### Methods and data collection

As previously reported [17,20], the Italian Association of Clinical Diabetologists (Associazione Medici Diabetologi, AMD) initiative and the relative database was established in order to identify a set of indicators that can be used in the context of continuous quality improvement. Participating centres adopted the same software systems for everyday management of outpatients, while a specially developed software package allowed us to extract the information we intended to analyze from all the clinical databases (AMD Data File). Moreover, data from all participating centers were collected and centrally analyzed anonymously [17,20].

This initiative includes measuring and monitoring HbA1c, BP, low-density lipoprotein (LDL-c), total and high density lipoprotein cholesterol (HDL-c) and triglycerides. The use of specific classes of drugs (insulin, statins and two or more anti-hypertensive agents) was also evaluated. Since normal ranges for HbA1c varied among centers, the percentage change with respect to the upper normal value (measured value⁄upper normal limit) was estimated and multiplied by 6.0 in order to allow comparisons among the centers. Kidney function was assessed by serum creatinine and urinary albumin excretion measurements. GFR was estimated for each patient using a standardized serum creatinine assay and the Chronic Kidney Disease Epidemiology Collaboration formula [21]. Increased urinary albumin excretion (i.e. albuminuria) was diagnosed if urinary albumin concentration was >30 mg/l, or if urinary albumin excretion rate was >20 μg/min, or if urinary albumin-to-creatinine ratio (ACR) was >2.5 mg/mmol in men and >3.5 mg/mmol in women.

At each participating centre all patients underwent physical examination and BP measurements according a standardized protocol. BP was measured with the patient in the sitting position after a 5-minute rest, with a mercury sphygmomanometer. Systolic BP and diastolic BP were read to the nearest 2 mmHg. Disappearance of Korotkoff sounds (phase V) was the criterion for diastolic BP. Three measurements were taken at 2-minute intervals and the average value was used to define clinical systolic BP and diastolic BP.

#### Metabolic syndrome

The revised definitions of the National Cholesterol Education Program-Adult Treatment Panel III, which suggests using BMI to define metabolic syndrome when waist circumference data are not readily available has been used in the present study. Accordingly, we did consider BMI ≥30 kg/m^2^ as a risk factor, because this value approximates a waist circumference >102 cm in men and >88 cm in women Since all study patients had T2D, Metabolic syndrome was defined as the presence of two or more of the following components: BMI ≥30 kg/m^2^, serum triglycerides ≥150 mg/dl or on treatment with fibrates, HDL <50 mg/dl in women and <40 mg/dl in men, hypertension (BP ≥130/85 mmHg or on antihypertensive medications) [22].

As expected mean serum uric acid (SUA) levels were significantly higher in men as compared to women (5.3 ±1.5 vs 4.8±1.7, P < 0.001). Therefore, we analyzed the study cohort on the basis of gender specific quintiles of SUA. Hyperuricemia (HU) was defined as the presence of SUA values in the range of the top gender specific SUA quintile (>5.8 mg/dL if female and >6.4 mg/dL if male).

CKD was defined as albuminuria or low eGFR (i.e. <60 mL/min/1.73 m^2^) or both.

Each patient have been followed- up for 4 years, underwent multiple regular visits within this timeframe Data on blood pressure, albuminuria and eGFR were collected on a yearly basis over the entire study period. In case a given patient reached any of the pre-specified renal end point before end of study, only data collected before the onset of that specific endpoint were considered.

### Renal outcomes

The outcomes were: i. eGFR <60 mL/min/1.73 m^2^; ii. Albuminuria; a composite of either eGFR <60 mL/min/1.73 m^2^ or Albuminuria.

### Exposure

The main analysis aimed at evaluating the association between baseline presence of Mets, its components, and HU and the incidence of renal outcomes during the study period.

### Statistical analysis

Data are given as mean values ± standard deviation (SD); categorical variables are described as frequencies and percentages. Variables associated to renal outcomes were evaluated by using logistic regression mixed models with diabetes clinics fitted as random effect to consider possible differences in data across centers. Odds ratios (ORs) were reported with their 95% confidence intervals (95% CIs). Multivariate models were fitted including patients with all data available. The analyses were made using STATA software, Version 14 (StataCorp, College Station, Texas). P values of <0.05 were considered statistically significant.

## 2. Results

Main clinical features of the study cohort at baseline, overall and on the basis of the presence/absence of Mets and HU, are summarized in Table 1. The mean age was 63±10 years, 56% of patients were males and the mean duration of diabetes was 10±8 years. By study design all patients had normal urine albumin excretion, eGFR was 87±13 mL/min/1.73m^2^. Mets was present in 59% (n = 8408) and HU in 18% (n = 2584) of patients, the average BP was 137±17/80±9 mmHg, with 88% of patients showing either systolic or diastolic values above 130/85 mmHg or BP treatment, the average HDL was 52±15 mg/dL, with 29% of patients showing HDL < 40 if male or < 50 mg/dL if female, the average triglycerides was 136±92 mg/dL with 31% of patients showing triglycerides ≥150 mg/dL or on fibrates, the average BMI was 29±5 Kg/m^2^ with 38% of patients with BMI ≥ 30 Kg/m^2^ at the baseline visit. Patients with baseline Mets and/or HU had a worse risk profile, namely higher BMI, BP values and a more unfavourable lipid profile despite lower disease duration and greater burden of antihypertensive and lipid lowering treatment.

**Table 1:** Clinical characteristics of study patients on the basis of the presence/ absence of metabolic syndrome and hyperuricemia (top gender-specific quintile).

Metabolic syndrome		No	Yes	No	Yes
SUA in the top gender-specific quintile		No	No	Yes	Yes
	All patients n = 14267	n = 5179	n = 6504	n = 680	n = 1904
Metabolic syndrome	8408 (58.9%)	-	-	-	-
BP≥130/85 or BP treatment	12509 (87.7%)	3895 (75.2%)	6219 (95.6%)	558 (82.1%)	1837 (96.5%)
Low HDL	4199 (29.4%)	174 (3.4%)	3071 (47.2%)	14 (2.1%)	940 (49.4%)
Triglycerides≥150 mg-dl or fibrates	4430 (31.1%)	172 (3.3%)	3204 (49.3%)	28 (4.1%)	1026 (53.9%)
BMI>30 Kg/m^2^	5489 (38.5%)	147 (2.8%)	3969 (61.0%)	18 (2.6%)	1355 (71.2%)
Only diabetes	853 (6.0%)	791 (15.3%)	0 (0%)	62 (9.1%)	0 (0%)
Diabetes and 1 factor	5006 (35.1%)	4388 (84.7%)	0 (0%)	618 (90.9%)	0 (0%)
Diabetes and 2 factors	4633 (32.5%)	0 (0%)	3758 (57.8%)	0 (0%)	875 (46.0%)
Diabetes and 3 factors	2745 (19.2%)	0 (0%)	2037 (31.3%)	0 (0%)	708 (37.2%)
Diabetes and 4 factors	1030 (7.2%)	0 (0%)	709 (10.9%)	0 (0%)	321 (16.9%)
SUA (mg/dL)	5.1±1.6	4.5±1	4.8±.9	7.2±3.1	7.1±1.8
SUA in the top gender-specific quintile	2584 (18.1%)	0 (0%)	0 (0%)	680 (100%)	1904 (100%)
Male sex	8040 (56.4%)	3206 (61.9%)	3381 (52.0%)	465 (68.4%)	988 (51.9%)
Age (years)	63±10	63±11	63±9	65±9	63±9
Known duration of diabetes (years)	10±8	11±9	9±8	9±8	8±7
BMI (Kg/m^2^)	29±5	26±3	31±5	27±2	33±5
Serum creatinine (mg/dL)	0.83±0.16	0.83±0.16	0.82±0.16	0.9±0.17	0.88±0.17
eGFR (mL/min/1.73 m^2^)	87±13	88±13	88±13	82±13	82±14
HbA1c (%)	7.2±1.3	7.1±1.2	7.4±1.3	6.8±1	7.1±1.1
HbA1c≥7%	7535 (53.2%)	2646 (51.3%)	3712 (57.6%)	254 (37.9%)	923 (48.9%)
Total cholesterol (mg/dL)	189±37	188±35	188±38	186±37	192±38
Triglycerides (mg/dL)	136±92	95±42	160±105	102±36	177±109
HDL (mg/dL)	52±15	59±14	47±14	57±13	46±13
LDL (mg/dL)	111±33	111±32	111±33	109±33	113±34
LDL ≥100 mg/dL	8808 (62.2%)	3255 (62.9%)	3964 (61.5%)	405 (59.9%)	1184 (63.4%)
Systolic BP (mmHg)	137±17	134±18	139±17	135±18	139±17
Diastolic BP (mmHg)	80±9	78±8	81±9	78±9	81±9
BP≥140/85 mmHg	7928 (55.6%)	2427 (46.9%)	4031 (62.0%)	339 (49.9%)	1131 (59.4%)
Retinopathy	2643 (18.5%)	963 (18.6%)	1264 (19.4%)	106 (15.6%)	310 (16.3%)
Smokers	1631 (17.2%)	603 (17.1%)	801 (18.8%)	53 (11.7%)	174 (14.2%)
Lipid-lowering treatment	6585 (46.2%)	2093 (40.4%)	3219 (49.5%)	302 (44.4%)	971 (51.0%)
Treatment with statins	6035 (42.3%)	2052 (39.6%)	2844 (43.7%)	290 (42.6%)	849 (44.6%)
Treatment with fibrates	321 (2.2%)	7 (.1%)	255 (3.9%)	1 (0.1%)	58 (3.0%)
Antihypertensive treatment	8961 (62.8%)	2461 (47.5%)	4551 (70.0%)	435 (64.0%)	1514 (79.5%)
Treatment with ACE-Is/ARBs	7464 (52.3%)	2009 (38.8%)	3808 (58.5%)	358 (52.6%)	1289 (67.7%)
Aspirin	4101 (28.7%)	1368 (26.4%)	1944 (29.9%)	193 (28.4%)	596 (31.3%)
*Antidiabetic therapy*					
Diet	1547 (10.8%)	652 (12.6%)	581 (8.9%)	116 (17.1%)	198 (10.4%)
Oral antidiabetic drugs	9563 (67.0%)	3256 (62.9%)	4459 (68.6%)	455 (66.9%)	1393 (73.2%)
Oral antidiabetic drugs and insulin	1726 (12.1%)	544 (10.5%)	925 (14.2%)	55 (8.1%)	202 (10.6%)
Insulin	1431 (10.0%)	727 (14.0%)	539 (8.3%)	54 (7.9%)	111 (5.8%)

Mean±SD or absolute frequency (percentage). ACE-Is, angiotensin converting enzyme-inhibitors; ARBs, angiotensin II receptor antagonists; BMI, body mass index; BP, blood pressure; eGFR, estimated glomerular filtration rate; HbA1c, glycated haemoglobin; HDL, high-density lipoprotein cholesterol; LDL, low-density lipoprotein cholesterol; SUA, serum uric acid; Gender specific highest quintile according to the baseline serum uric acid levels: 5.8 mg/dL in females and 6.4 mg/dL in males). Patients' baseline missing data: known duration of diabetes in 212 (1.5%), HbA1c in 115 (0.8%), total cholesterol in 21 (0.1%), and smoking status in 4808 (33.7%).

Baseline clinical features of patients grouped on the basis of SUA levels and individual components of Mets are reported in S1 Table.

Patients in the top gender specific SUA quintile (n = 2,584) showed a worse clinical and metabolic profile at baseline. They were older, had higher BMI, BP values, lower GFR values and a worse lipid profile. They were more likely to have Mets (74% vs 56%, p<0.001) and a good glycemic control despite a lower prevalence of insulin treatment.

As expected, despite being more frequently females, patients with Mets (n = 8408) showed higher BP values, an unfavorable metabolic profile (included higher HbA1c) and significantly higher SUA levels (5.3±1.5 vs 4.8±1.6, P<0.001) as compared to those without Mets. There was no difference in age and eGFR on the basis of presence/absence of Mets.

Being in the top gender-specific uric acid quintile (HU) doubled the risk of having MS (OR 2.32 95% CI 2.10–2.56, P<0.001) even after adjusting for several variables, including HbA1c, known duration of diabetes and eGFR.

Within 4-year follow-up, 1,990 (13.9%) patients developed eGFR <60 mL/min/1.73 m^2^, 3,740 (26.2%) albuminuria, and 5,041 (35%) CKD (i.e., eGFR <60 mL/min/1.73 m^2^ and/or albuminuria). In S2 and S3 Figs. renal outcomes are reported as stratified by number of Mets components and presence of Mets. There was a progressive increase of incident low eGFR (i.e. eGFR<60 mL/min/1.73m2) and albuminuria across the five subgroups (from 5.7 to 17.6%, and from 17.8 to 30.7%, 0 vs. 4 components beyond diabetes, respectively). Patients with Mets at baseline developed reduced eGFR and albuminuria more frequently as compared to those without Mets (15.6 vs 11.6% and 28.2 vs 23.4%, p<0.001 respectively). Moreover, patients with HU at baseline developed reduced eGFR and albuminuria more frequently as compared to those without HU (20.9 vs 12.4% and 30 vs 25.4%, p<0.001 respectively).

At univariate analysis, patients with each single Mets component or with HU reached low eGFR and albuminuria significantly more frequently than those without it (S2 Table). Among different components of the Mets, BP had the greatest impact on the incidence of measures of CKD (OR 3.05 95% CI 2.46–3.77, P<0.001 for low eGFR and OR 1.62 95% CI 1.41–1.85, P<0.001 for albuminuria, respectively).

The independent association of several variables with renal outcomes at four year follow-up, was further investigated by logistic multivariate analysis among all and separately for males and females. In the general cohort and in males, SUA levels and metabolic components (with the exception of diastolic BP) strongly and significantly predicted the development of low eGFR and albuminuria, independently of a number of potential confounders, included baseline eGFR and HbA1c (S3 Table). Among females, the risk for the onset of low eGFR was significantly and independently associated to BMI and SUA levels at baseline, while the risk for the incidence of albuminuria was linked with a decrease in diastolic BP and HDL values further than with an increase in BMI and SUA levels. We further examined the associations of HU and individual components of Mets and the risk for new onset of low eGFR and albuminuria in a similar model among all with a dichotomous approach: the presence of BP ≥130/85, low HDL, Triglicerydes ≥150, BMI ≥30 and HU independently increased the risk for eGFR <60 ml/min/1.73m^2^ (1.57, P<0.001; 1.13, P = 0.056; 1.18, P = 0.008; 1.21, P0.002; 1.26, P = 0.001) and for albuminuria (1.35, P<0.001; 1.18, P = 0.001; 1.15, P = 0.002; 1.24, P = 0.001; 1.17, P = 0.004) respectively. Finally, when Mets (and not its components) was included in the model, the presence of Mets and of HU, independently predicted the development of eGFR (1.41, P<0.001; 1.29, P<0.001, respectively) and albuminuria (1.33, P<0.001; 1.21, P<0.001, respectively).

**Fig 1:**
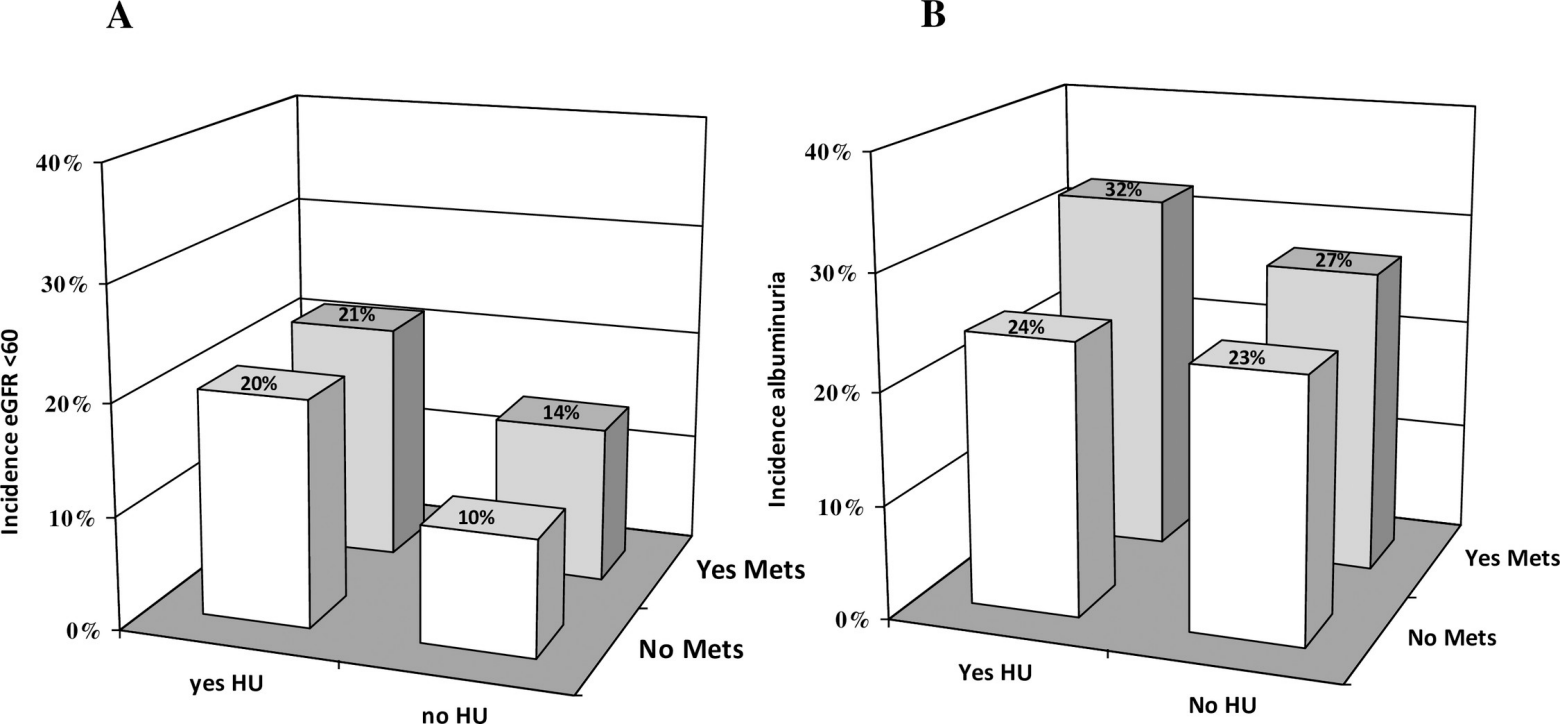
1A. Incidence of GFR reduction on the basis of the presence/absence of metabolic syndrome and hyperuricemia (top gender-specific quintile)1B. Incidence of albuminuria on the basis of the presence/absence of metabolic syndrome and hyperuricemia (top gender-specific quintile)

We performed further analyses after classifying patients into four sub-groups on the basis of the presence/absence of HU and MS (Fig 1A and 1B, Table 2). Fig 1A and 1B show the incidence of GFR reduction and albuminuria according to this stratification. The incidence of low eGFR was higher for patients with HU independent of the presence or absence of Mets as compared to those without HU and Mets (21% and 20% vs 10.4%, p<0.001 respectively). The incidence of albuminuria was higher for patients with Mets and HU as compared to reference (those without HU and Mets) (32 vs 23%, p<0.001). In those without Mets, HU did not influence the risk to develop albuminuria.

**Table 2:** Determinants of measures of renal outcome including the presence/absence of Metabolic Syndrome and/or Hyperuricemia.

	eGFR <60 mL/min/1.73m^2^		Albuminuria	
	OR (95%CI)	p	OR (95%CI)	p
1HU-Mets-	*Reference*	*-*	*Reference*	*-*
2HU-Mets+	1.16 (0.99–1.36)	0.069	1.02 (0.91–1.14)	0.766
3HU+Mets-	1.57 (1.23–1.99)	<0.001	1.00 (0.81–1.22)	0.964
4HU+Mets+	1.30 (1.07–1.59)	0.009	1.25 (1.07–1.46)	0.005
Systolic BP (by 10 mmHg)	1.07 (1.03–1.11)	<0.001	1.06 (1.03–1.09)	<0.001
Diastolic BP (by 10 mmHg)	0.92 (0.85–0.99)	0.034	0.96 (0.90–1.02)	0.164
HDL (by 5 mg/dL)	0.97 (0.95–0.99)	0.006	0.96 (0.95–0.98)	<0.001
Triglycerides (by 50 mg/dL)	1.06 (1.03–1.09)	<0.001	1.03 (1.01–1.05)	0.013
BMI (by 5 Kg/m^2^)	1.11 (1.04–1.19)	0.002	1.10 (1.05–1.16)	<0.001
Male sex	0.76 (0.67–0.85)	<0.001	1.37 (1.25–1.49)	<0.001
Age (by 10 years)	1.53 (1.41–1.65)	<0.001	1.19 (1.13–1.26)	<0.001
Known duration of diabetes (by 10 years)	1.11 (1.03–1.18)	0.003	1.02 (0.97–1.08)	0.398
eGFR (by 10 mL/min/1.73 m^2^)	0.40 (0.38–0.42)	<0.001	1.01 (0.97–1.04)	0.791
HbA1c (by 1%)	1.12 (1.07–1.18)	<0.001	1.12 (1.08–1.16)	<0.001

Multivariate models. BMI, body mass index; BP, blood pressure; eGFR, estimated glomerular filtration rate; HbA1c, glycated haemoglobin; HDL, high-density lipoprotein cholesterol; HU, serum uric acid in the top gender-specific quintile; LDL, low-density lipoprotein cholesterol; Mets, Metabolic syndrome. Complete-case analysis performed excluding 273 patients with incomplete data.

Having HU was the stronger independent risk factor for the development of low eGFR with an OR ranging from 1.3 to 1.57 according to the presence of Mets or not. Furthermore, HU showed a significant, additive role to the presence of Mets in the risk for the onset of albuminuria which increased significantly (by 25%) only for patients with Mets and HU as compared to those without Mets and HU (95% CI 1.07–1.46, P <0.001) (Table 2).

## 3. Discussion

Our data provide new insights into the complex relationship between SUA, Mets and the incidence of CKD in a large longitudinal cohort study of T2D patients treated in accordance with evidence based guidelines.

Patients with HU were 2 times more likely to concomitantly show Mets. The odds of developing eGFR <60 ml/min per 1.73 m^2^ and albuminuria during follow-up increased along with the number of components of Mets from 0 to 4 beyond diabetes. Furthermore, Mets and HU emerged as independent risk factors for the development of both low eGFR and albuminuria; the former was more likely to occur in the presence of HU at baseline, whereas the incidence of albuminuria was significantly increased only in those patients simultaneously presenting HU and Mets at baseline.

To the best of our knowledge, this is the first time that longitudinal data on the association between Mets and CKD are reported in a non-Asiatic cohort of patients with T2D. Our 41% incidence and 33% increased risk for development of low eGFR and albuminuria respectively among patients with Mets (Fig 1) is in accordance with data from the literature [7]. In particular, the only prospective study published so far investigating the relationship between Mets and incidence of CKD in 5,829 Chinese patients with T2D found a 31% higher risk of renal outcome for subjects with Mets after a median follow-up duration of 4.6 years [9]. CKD was defined as the first eGFR<60 ml/min per 1.73 m^2^ or the first hospitalization with CKD event, adjudicated on the basis of hospital discharge diagnoses coded by the ICD-9.

Among the small number of prospective studies investigating the risk for developing either microalbuminuria or dipstick-positive proteinuria among patients with Mets in non diabetic patients [10–14], three studies showed a positive association, reporting a higher risk for proteinuria in patients with Mets independently of gender [12, 14] or in men only [11].

An increase in SUA levels has repeatedly been demonstrated to be a risk factor for the development of T2D [23, 24], and therefore the better glycemic control we describe among patients with HU, as compared to those without it, seems counterintuitive (S1 Table) and deserves further comment. As a matter of fact, an inverse correlation between SUA and HbA1c levels has already been described in previous cross sectional studies on US and Chinese populations [25, 26] and might be justified by a larger urine loss of uric acid in exchange for a greater tubular handling of glucose due to glycosuria [27]. In fact, as suggested by studies on patients with type 1 diabetes [28], a larger amount of glycosuria in patients with poor glycemic control can lower SUA by decreasing uric acid reabsorption at the tubular level. Although the molecular mechanisms underlying the uricosuric effect of glucose has not been completely elucidated, interaction between glucose and urate transporters at the tubule level is highly plausible and might result in less renal uric acid reabsorption and lower serum levels [29].

On the contrary, higher insulin levels are known to reduce renal excretion of urate in both healthy and hypertensive subjects [30], providing a strong mechanism supporting the previously described link between the Mets and HU [31, 32] and confirmed in the present cohort where 74% of HU patients showed Mets (S1 Table). Interestingly, high SUA concentrations commonly precede the development of insulin resistance in experimental studies [33, 34] and frequently forerun the diagnosis of Mets in clinical reports [31, 35]. This has led several authors to suggest that hyperuricemia may be a risk factor and a new marker for Mets. Data presented herein support a role for SUA as a promoter of CKD, which in turn is a clinical condition frequently associated to Mets [36]. In particular, our data suggest that relationship between SUA values and both MS as well as the incidence of CKD extends even below the traditional cut-off used for hyperuricemia [37,38].

We observed increased ORs for new onset CKD along with the increasing number of baseline MS components in agreement with previous reports [39, 40]. Hypertension is regarded as the leading risk factor for development and progression of CKD in non-diabetic and diabetic individuals and this is supported by our data showing that BP is the main risk factor for CKD at univariate analysis. In fact, patients with BP >130/80 or antihypertensive treatment showed a 3 times higher risk to develop low eGFR as compared to those without increased BP values. Accordingly, after full adjustment, this single Mets component showed to be the strongest contributor to the development of CKD increasing by as much as 57 and 35% the incidence of low eGFR and albuminuria respectively during follow-up.

While there are strong experimental and clinical data supporting the independent role of increased SUA levels as a promoter of progression and development of CKD in T2D [16, 17], the interaction between SUA and Mets has been much less investigated so far [32]. In this context, our data indicate that SUA impact on renal outcomes is independent of the presence of Mets and its components, thus supporting a link between Mets and renal damage at least in part independent of hyperinsulinemia and its associates. To our knowledge, this is the first prospective study to investigate the combined role of Mets and HU on the incidence of CKD components. HU increased significantly the risk of developing low eGFR in our study patients with and without Mets, whereas the risk of albuminuria significantly increased only when Mets was associated with HU (Table 2). Moreover, patients with both HU and Mets and patients with either one of these risk factors showed greater eGFR reduction (adjusted for baseline eGFR) over the entire study period as compared to those without HU and Mets (p<0.01).

These findings highlight the detrimental interaction of SUA and Mets at least in T2D.

In addition to high BP and hyperglycemia, several pathophysiological mechanism, including insulin resistance, inflammation, oxidative stress, endothelial dysfunction, increased fructose intake, increased cytokine and adipokine synthesis and abnormalities in the sympathetic nervous system, have all been suggested as important factors mediating glomerular and tubular fibrosis and vascular disease described in patients with Mets [41, 42]. While more studies are needed to prove causality, these pathogenetic mechanisms support our findings of an increased burden of renal risk in patients with T2D and Mets. Furthermore, our results add to the existing body of literature about the harmful effects of Mets on the development of renal damage and support the hypothesis that the frequent coexistence of Mets and HU might explain part of the pathophysiological link between Mets and CKD in T2D.

Our study has some limitations as well as several strengths that should be mentioned. The lack of data about waist circumference is a major limitation of the present analysis, especially when one considers that in the presence of diabetes, low BMI may reflect lipolysis and muscle wasting due to hyperglycemia and insufficient insulin action. Furthermore, the prognostic information provided by BMI has been shown to vary depending on body composition and clinical settings [43]. Although our analyses took into account several confounding variables, we lacked data relating to physical activity, diet, socioeconomic status, diagnosis of gout and family history of cardiovascular and kidney disease which might have influenced the results. Furthermore, we did not gather specific information on urate lowering treatment. This, however, does not lessen the core message of our study, which deals with the prognostic power of SUA values independent of confounding variables.

Moreover, we must acknowledge that laboratory parameters, including serum creatinine were not measured in a centralized laboratory and this may have led to some variability, especially in GFR estimation. In addition, information on albuminuria has been gathered only as a categorical trait and this may also have contributed to variability in the outcome measure.

On the other hand, the large size and homogeneous clinical characteristics of our study cohort as well as the representative geographical distribution of the recruiting centers certainly contribute to make our results a good representation of real life clinical condition.

## 4. Conclusions

In conclusion, Mets, its components and HU entail an increased risk for developing kidney disease in patients with T2D. These results may be relevant to help focusing on better prevention and therapeutic strategies as epidemiological surveys indicate increasing prevalence of both Mets and HU and, accordingly, the burden of chronic renal disease remain a major health concern in T2D.

## Supporting information

S1 Table (DOCX)

S2 Table (DOCX)

S3 Table (DOCX)

S1 Fig Flow diagram for selection of study patients. (TIFF)

S2 Fig Incidence of GFR reduction on the basis of the presence/ absence of metabolic syndrome and the number of its components. (TIFF)

S3 Fig Incidence of albuminuria on the basis of the presence/ absence of metabolic syndrome and the number of its components. (TIF)

S1 File AMD ANNALS Study Group and Participating Centers. (PDF)
