# Supplementary material for: Metabolic syndrome, serum uric acid and renal risk in patients with T2D
Source: PLoS One. 2017 Apr 19;12(4):e0176058. doi: 10.1371/journal.pone.0176058 (PMC5396926; doi:10.1371/journal.pone.0176058)
Supplement: S1 Table — (DOCX) [file pone.0176058.s001.docx]

**S1 Table**

**Clinical characteristics of study patients by uric acid levels and individual components of metabolic syndrome**

|  | **SUA top quintile** | | **BP≥130/85 or BP treatment** | | **Low HDL** | | **Triglycerides≥150 mg/dl** | | **BMI>30 Kg/m^2^** | |
| --- | --- | --- | --- | --- | --- | --- | --- | --- | --- | --- |
|  | **No** | **Yes** | **No** | **Yes** | **No** | **Yes** | **No** | **Yes** | **No** | **Yes** |
|  | **n=11683** | **n=2584** | **n=1758** | **n=12509** | **n=10068** | **n=4199** | **n=9837** | **n=4430** | **n=8778** | **n=5489** |
| Metabolic syndrome | 6504 (55.7%) | 1904 (73.7%) | 352 (20.0%) | 8056 (64.4%) | 4397 (43.7%) | 4011 (95.5%) | 4178 (42.5%) | 4230 (95.5%) | 3084 (35.1%) | 5324 (97.0%) |
| BP≥130/85 or BP treatment | 10114 (86.6%) | 2395 (92.7%) | 0 (0%) | 12509 (100%) | 8789 (87.3%) | 3720 (88.6%) | 8556 (87.0%) | 3953 (89.2%) | 7394 (84.2%) | 5115 (93.2%) |
| Low HDL | 3245 (27.8%) | 954 (36.9%) | 479 (27.2%) | 3720 (29.7%) | 0 (0%) | 4199 (100%) | 2100 (21.3%) | 2099 (47.4%) | 2192 (25.0%) | 2007 (36.6%) |
| Triglycerides≥150 mg-dl or fibrates | 3376 (28.9%) | 1054 (40.8%) | 477 (27.1%) | 3953 (31.6%) | 2331 (23.2%) | 2099 (50.0%) | 0 (0%) | 4430 (100%) | 2276 (25.9%) | 2154 (39.2%) |
| BMI>30 Kg/m^2^ | 4116 (35.2%) | 1373 (53.1%) | 374 (21.3%) | 5115 (40.9%) | 3482 (34.6%) | 2007 (47.8%) | 3335 (33.9%) | 2154 (48.6%) | 0 (0%) | 5489 (100%) |
| Only diabetes | 791 (6.8%) | 62 (2.4%) | 853 (48.5%) | 0 (0%) | 853 (8.5%) | 0 (0%) | 853 (8.7%) | 0 (0%) | 853 (9.7%) | 0 (0%) |
| Diabetes and 1 factor | 4388 (37.6%) | 618 (23.9%) | 553 (31.5%) | 4453 (35.6%) | 4818 (47.9%) | 188 (4.5%) | 4806 (48.9%) | 200 (4.5%) | 4841 (55.1%) | 165 (3.0%) |
| Diabetes and 2 factors | 3758 (32.2%) | 875 (33.9%) | 279 (15.9%) | 4354 (34.8%) | 3407 (33.8%) | 1226 (29.2%) | 3349 (34.0%) | 1284 (29.0%) | 2231 (25.4%) | 2402 (43.8%) |
| Diabetes and 3 factors | 2037 (17.4%) | 708 (27.4%) | 73 (4.2%) | 2672 (21.4%) | 990 (9.8%) | 1755 (41.8%) | 829 (8.4%) | 1916 (43.3%) | 853 (9.7%) | 1892 (34.5%) |
| Diabetes and 4 factors | 709 (6.1%) | 321 (12.4%) | 0 (0%) | 1030 (8.2%) | 0 (0%) | 1030 (24.5%) | 0 (0%) | 1030 (23.3%) | 0 (0%) | 1030 (18.8%) |
| SUA (mg/dL) | 4.6±1.0 | 7.1±2.2 | 4.7±1.5 | 5.2±1.6 | 5.0±1.6 | 5.2±1.5 | 5.0±1.6 | 5.3±1.6 | 4.9±1.6 | 5.4±1.5 |
| SUA in the top gender-specific quintile | 0 (0%) | 2584 (100%) | 189 (10.8%) | 2395 (19.1%) | 1630 (16.2%) | 954 (22.7%) | 1530 (15.6%) | 1054 (23.8%) | 1211 (13.8%) | 1373 (25.0%) |
| Male sex | 6587 (56.4%) | 1453 (56.2%) | 1044 (59.4%) | 6996 (55.9%) | 6097 (60.6%) | 1943 (46.3%) | 5580 (56.7%) | 2460 (55.5%) | 5341 (60.8%) | 2699 (49.2%) |
| Age (years) | 63±10 | 64±9 | 56±12 | 64±9 | 64±10 | 62±10 | 64±10 | 62±10 | 63±10 | 62±9 |
| Known duration of diabetes (years) | 10±8 | 9±8 | 8±8 | 10±8 | 10±8 | 9±8 | 10±9 | 9±7 | 10±8 | 9±8 |
| BMI (Kg/m^2^) | 29±5 | 31±5 | 27±4 | 30±5 | 29±5 | 31±5 | 29±5 | 31±5 | 26±3 | 34±4 |
| Serum creatinine (mg/dL) | 0.82±0.16 | 0.88±0.17 | 0.83±0.16 | 0.83±0.16 | 0.84±0.16 | 0.82±0.16 | 0.83±0.16 | 0.84±0.17 | 0.84±0.16 | 0.82±0.16 |
| eGFR (mL/min/1.73 m^2^) | 88±13 | 82±13 | 92±14 | 86±13 | 86±13 | 87±14 | 87±13 | 86±14 | 87±13 | 87±13 |
| HbA1c (%) | 7.3±1.3 | 7.0±1.1 | 7.2±1.4 | 7.2±1.3 | 7.2±1.2 | 7.3±1.4 | 7.1±1.2 | 7.5±1.4 | 7.2±1.2 | 7.3±1.3 |
| HbA1c≥7% | 6358 (54.8%) | 1177 (46%) | 907 (51.8%) | 6628 (53.4%) | 5227 (52.3%) | 2308 (55.4%) | 4927 (50.4%) | 2608 (59.5%) | 4455 (51.1%) | 3080 (56.6%) |
| Total cholesterol (mg/dL) | 188±37 | 191±38 | 192±38 | 188±37 | 192±36 | 180±38 | 183±35 | 200±39 | 188±37 | 189±37 |
| Triglycerides (mg/dL) | 131±89 | 158±101 | 129±98 | 137±91 | 120±76 | 174±114 | 96±28 | 224±119 | 126±85 | 151±101 |
| HDL (mg/dL) | 52±15 | 49±14 | 52±16 | 51±15 | 58±13 | 37±9 | 54±15 | 45±12 | 53±16 | 50±14 |
| LDL (mg/dL) | 111±33 | 112±34 | 116±33 | 111±33 | 111±32 | 110±34 | 110±32 | 113±35 | 111±33 | 111±33 |
| LDL ≥100 mg/dL | 7219 (62.2%) | 1589 (62.5%) | 1173 (67.3%) | 7635 (61.5%) | 6302 (62.8%) | 2506 (60.7%) | 6049 (61.6%) | 2759 (63.7%) | 5432 (62.3%) | 3376 (62%) |
| Systolic BP (mmHg) | 137±17 | 138±17 | 116±7 | 140±16 | 137±17 | 136±17 | 137±17 | 137±17 | 136±17 | 139±17 |
| Diastolic BP (mmHg) | 79±9 | 80±9 | 73±7 | 81±8 | 80±9 | 80±9 | 79±9 | 80±9 | 79±9 | 81±9 |
| BP≥140/85 mmHg | 6458 (55.3%) | 1470 (56.9%) | 0 (0%) | 7928 (63.4%) | 5631 (55.9%) | 2297 (54.7%) | 5421 (55.1%) | 2507 (56.6%) | 4509 (51.4%) | 3419 (62.3%) |
| Retinopathy | 2227 (19.1%) | 416 (16.1%) | 247 (14.1%) | 2396 (19.2%) | 1886 (18.7%) | 757 (18.0%) | 1925 (19.6%) | 718 (16.2%) | 1598 (18.2%) | 1045 (19.0%) |
| Smokers | 1404 (18.0%) | 227 (13.5%) | 314 (26.2%) | 1317 (15.9%) | 1059 (15.5%) | 572 (21.6%) | 993 (15.2%) | 638 (21.7%) | 1085 (18.6%) | 546 (15.1%) |
| Lipid-lowering treatment | 5312 (45.5%) | 1273 (49.3%) | 571 (32.5%) | 6014 (48.1%) | 4495 (44.6%) | 2090 (49.8%) | 4169 (42.4%) | 2416 (54.5%) | 3998 (45.5%) | 2587 (47.1%) |
| Treatment with statins | 4896 (41.9%) | 1139 (44.1%) | 504 (28.7%) | 5531 (44.2%) | 4243 (42.1%) | 1792 (42.7%) | 4108 (41.8%) | 1927 (43.5%) | 3691 (42.0%) | 2344 (42.7%) |
| Treatment with fibrates | 262 (2.2%) | 59 (2.3%) | 33 (1.9%) | 288 (2.3%) | 147 (1.5%) | 174 (4.1%) | 0 (0%) | 321 (7.2%) | 173 (2.0%) | 148 (2.7%) |
| Antihypertensive treatment | 7012 (60.0%) | 1949 (75.4%) | 0 (0%) | 8961 (71.6%) | 6167 (61.3%) | 2794 (66.5%) | 6046 (61.5%) | 2915 (65.8%) | 4935 (56.2%) | 4026 (73.3%) |
| Treatment with ACE-Is/ARBs | 5817 (49.8%) | 1647 (63.7%) | 0 (0%) | 7464 (59.7%) | 5116 (50.8%) | 2348 (55.9%) | 5035 (51.2%) | 2429 (54.8%) | 4054 (46.2%) | 3410 (62.1%) |
| Aspirin | 3312 (28.3%) | 789 (30.5%) | 223 (12.7%) | 3878 (31.0%) | 2920 (29.0%) | 1181 (28.1%) | 2873 (29.2%) | 1228 (27.7%) | 2440 (27.8%) | 1661 (30.3%) |
| *Antidiabetic therapy* |  |  |  |  |  |  |  |  |  |  |
| Diet | 1233 (10.6%) | 314 (12.2%) | 253 (14.4%) | 1294 (10.3%) | 1157 (11.5%) | 390 (9.3%) | 1124 (11.4%) | 423 (9.5%) | 1067 (12.2%) | 480 (8.7%) |
| Oral antidiabetic drugs | 7715 (66.0%) | 1848 (71.5%) | 1093 (62.2%) | 8470 (67.7%) | 6706 (66.6%) | 2857 (68.0%) | 6445 (65.5%) | 3118 (70.4%) | 5718 (65.1%) | 3845 (70.0%) |
| Oral antidiabetic drugs and insulin | 1469 (12.6%) | 257 (9.9%) | 176 (10.0%) | 1550 (12.4%) | 1149 (11.4%) | 577 (13.7%) | 1151 (11.7%) | 575 (13.0%) | 940 (10.7%) | 786 (14.3%) |
| Insulin | 1266 (10.8%) | 165 (6.4%) | 236 (13.4%) | 1195 (9.6%) | 1056 (10.5%) | 375 (8.9%) | 1117 (11.4%) | 314 (7.1%) | 1053 (12.0%) | 378 (6.9%) |

Mean±SD or absolute frequency (percentage). ACE-Is, angiotensin converting enzyme-inhibitors; ARBs, angiotensin II receptor antagonists; BMI, body mass index; BP, blood pressure; eGFR, estimated glomerular filtration rate; HbA1c, glycated haemoglobin; HDL, high-density lipoprotein cholesterol; LDL, low-density lipoprotein cholesterol; SUA, serum uric acid; Gender specific highest quintile according to the baseline serum uric acid levels: 5.8 mg/dL in females and 6.4 mg/dL in males). Patients' baseline missing data: known duration of diabetes in 212 (1.5%), HbA1c in 115 (0.8%), total cholesterol in 21 (0.1%), and smoking status in 4808 (33.7%).
