# Supplementary material for: Metabolic syndrome, serum uric acid and renal risk in patients with T2D
Source: PLoS One. 2017 Apr 19;12(4):e0176058. doi: 10.1371/journal.pone.0176058 (PMC5396926; doi:10.1371/journal.pone.0176058)
Supplement: S2 Table — (DOCX) [file pone.0176058.s002.docx]

**S2 Table**

**Cumulative incidence of renal outcomes by uric acid levels and individual components of metabolic syndrome**

|  | **SUA top quintile** | | **BP≥130/85 or BP treatment** | | **Low HDL** | | **Triglycerides≥150 mg/dl** | | **BMI>30 Kg/m^2^** | |  |  |
| --- | --- | --- | --- | --- | --- | --- | --- | --- | --- | --- | --- | --- |
|  | **No** | **Yes** | **No** | **Yes** | **No** | **Yes** | **No** | **Yes** | **No** | **Yes** |  |  |
|  | **n=11683** | **n=2584** | **n=1758** | **n=12509** | **n=10068** | **n=4199** | **n=9837** | **n=4430** | **n=8778** | **n=5489** |  |  |
|  |  |  |  |  |  |  |  |  |  |  |  |  |
| *4-year outcome* |  |  |  |  |  |  |  |  |  |  |  |  |
| GFR<60 | 1449 (12.4%) | 541 (20.9%) | 97 (5.5%) | 1893 (15.1%) | 1368 (13.6%) | 622 (14.8%) | 1280 (13%) | 710 (16%) | 1148 (13.1%) | 842 (15.3%) |  |  |
| albU+ | 2964 (25.4%) | 776 (30%) | 346 (19.7%) | 3394 (27.1%) | 2546 (25.3%) | 1194 (28.4%) | 2483 (25.2%) | 1257 (28.4%) | 2143 (24.4%) | 1597 (29.1%) |  |  |
| DKD (GFR<60 OR albU+) | 3918 (33.5%) | 1123 (43.5%) | 416 (23.7%) | 4625 (37%) | 3460 (34.4%) | 1581 (37.7%) | 3339 (33.9%) | 1702 (38.4%) | 2901 (33%) | 2140 (39%) |  |  |
| GFR<60 AND albU+ | 495 (4.2%) | 194 (7.5%) | 27 (1.5%) | 662 (5.3%) | 454 (4.5%) | 235 (5.6%) | 424 (4.3%) | 265 (6%) | 390 (4.4%) | 299 (5.4%) |  |  |
|  |  |  |  |  |  |  |  |  |  |  |  |  |
|  |  |  |  |  |  |  |  |  |  |  |  |  |
|  |  |  |  |  |  |  |  |  |  |  |  |  |
|  |  |  |  |  |  |  |  |  |  |  |  |  |
|  |  |  |  |  |  |  |  |  |  |  |  |  |
|  |  |  |  |  |  |  |  |  |  |  |  |  |
|  |  |  |  |  |  |  |  |  |  |  |  |  |
|  |  |  |  |  |  |  |  |  |  |  | |  |
|  |  |  |  |  |  |  |  |  |  |  | |  |
|  |  |  |  |  |  |  |  |  |  |  |  |  |
|  |  |  |  |  |  |  |  |  |  |  |  |  |
|  |  |  |  |  |  |  |  |  |  |  |  |  |
|  |  |  |  |  |  |  |  |  |  |  |  |  |
|  |  |  |  |  |  |  |  |  |  |  |  |  |
|  |  |  |  |  |  |  |  |  |  |  |  |  |
|  |  |  |  |  |  |  |  |  |  |  |  |  |
|  |  |  |  |  |  |  |  |  |  |  |  |  |
|  |  |  |  |  |  |  |  |  |  |  |  |  |
|  |  |  |  |  |  |  |  |  |  |  |  |  |
|  |  |  |  |  |  |  |  |  |  |  |  |  |
|  |  |  |  |  |  |  |  |  |  |  |  |  |
|  |  |  |  |  |  |  |  |  |  |  |  |  |
|  |  |  |  |  |  |  |  |  |  |  |  |  |
|  |  |  |  |  |  |  |  |  |  |  |  |  |
|  |  |  |  |  |  |  |  |  |  |  |  |  |
|  |  |  |  |  |  |  |  |  |  |  |  |  |
|  |  |  |  |  |  |  |  |  |  |  |  |  |
|  |  |  |  |  |  |  |  |  |  |  |  |  |
|  |  |  |  |  |  |  |  |  |  |  |  |  |
|  |  |  |  |  |  |  |  |  |  |  |  |  |
|  |  |  |  |  |  |  |  |  |  |  |  |  |
|  |  |  |  |  |  |  |  |  |  |  |  |  |
|  |  |  |  |  |  |  |  |  |  |  |  |  |
|  |  |  |  |  |  |  |  |  |  |  |  |  |
|  |  |  |  |  |  |  |  |  |  |  |  |  |

Mean±SD or absolute frequency (percentage). ACE-Is, angiotensin converting enzyme-inhibitors; ARBs, angiotensin II receptor antagonists; BMI, body mass index; BP, blood pressure; eGFR, estimated glomerular filtration rate; HbA1c, glycated haemoglobin; HDL, high-density lipoprotein cholesterol; LDL, low-density lipoprotein cholesterol; SUA, serum uric acid; Gender specific highest quintile according to the baseline serum uric acid levels: 5.8 mg/dL in females and 6.4 mg/dL in males). Patients' baseline missing data: known duration of diabetes in 212 (1.5%), HbA1c in 115 (0.8%), total cholesterol in 21 (0.1%), and smoking status in 4808 (33.7%).
