# Supplementary material for: Metabolic syndrome, serum uric acid and renal risk in patients with T2D
Source: PLoS One. 2017 Apr 19;12(4):e0176058. doi: 10.1371/journal.pone.0176058 (PMC5396926; doi:10.1371/journal.pone.0176058)
Supplement: S3 Table — (DOCX) [file pone.0176058.s003.docx]

**S3 Table. Determinants of measures of renal outcome**

|  | **eGFR <60 mL/min/1.73m^2^** | | **Albuminuria** | |
| --- | --- | --- | --- | --- |
|  | **Odds Ratio (95%CI)** | **p** | **Odds Ratio (95%CI)** | **p** |
| **All patients (n=13994)** |  |  |  |  |
| Systolic BP (by 10 mmHg) | 1.07 (1.03-1.11) | <0.001 | 1.06 (1.03-1.09) | <0.001 |
| Diastolic BP (by 10 mmHg) | 0.92 (0.85-0.99) | 0.035 | 0.96 (0.90-1.02) | 0.183 |
| HDL (by 5 mg/dL) | 0.96 (0.94-0.98) | 0.001 | 0.96 (0.94-0.98) | <0.001 |
| Triglycerides (by 50 mg/dL) | 1.06 (1.03-1.09) | <0.001 | 1.03 (1.01-1.06) | 0.007 |
| BMI (by 5 Kg/m^2^) | 1.12 (1.06-1.19) | <0.001 | 1.11 (1.06-1.16) | <0.001 |
| SUA (by 1 mg/dL) | 1.07 (1.04-1.11) | <0.001 | 1.05 (1.02-1.07) | 0.001 |
| Male sex | 0.72 (0.64-0.81) | <0.001 | 1.32 (1.21-1.45) | <0.001 |
| Age (by 10 years) | 1.53 (1.41-1.65) | <0.001 | 1.19 (1.13-1.26) | <0.001 |
| Known duration of diabetes (by 10 years) | 1.11 (1.03-1.18) | 0.003 | 1.02 (0.97-1.08) | 0.390 |
| eGFR (by 10 mL/min/1.73 m^2^) | 0.40 (0.38-0.42) | <0.001 | 1.01 (0.97-1.05) | 0.697 |
| HbA1c (by 1%) | 1.13 (1.07-1.19) | <0.001 | 1.13 (1.09-1.17) | <0.001 |
|  |  |  |  |  |
| **Males (n=7871)** |  |  |  |  |
| Systolic BP (by 10 mmHg) | 1.11 (1.05-1.17) | <0.001 | 1.08 (1.03-1.12) | <0.001 |
| Diastolic BP (by 10 mmHg) | 0.92 (0.82-1.03) | 0.151 | 0.99 (0.92-1.07) | 0.853 |
| HDL (by 5 mg/dL) | 0.94 (0.91-0.97) | <0.001 | 0.96 (0.93-0.98) | <0.001 |
| Triglycerides (by 50 mg/dL) | 1.08 (1.03-1.12) | 0.001 | 1.04 (1.01-1.07) | 0.019 |
| BMI (by 5 Kg/m^2^) | 1.14 (1.03-1.26) | 0.010 | 1.13 (1.06-1.21) | <0.001 |
| SUA (by 1 mg/dL) | 1.09 (1.03-1.14) | 0.002 | 1.05 (1.01-1.09) | 0.007 |
| Age (by 10 years) | 1.56 (1.39-1.74) | <0.001 | 1.22 (1.13-1.31) | <0.001 |
| Known duration of diabetes (by 10 years) | 1.06 (0.96-1.17) | 0.221 | 0.99 (0.92-1.06) | 0.743 |
| eGFR (by 10 mL/min/1.73 m^2^) | 0.37 (0.34-0.40) | <0.001 | 0.99 (0.94-1.04) | 0.731 |
| HbA1c (by 1%) | 1.14 (1.06-1.22) | 0.001 | 1.11 (1.06-1.17) | <0.001 |
|  |  |  |  |  |
| **Females (n=6073)** |  |  |  |  |
| Systolic BP (by 10 mmHg) | 1.04 (0.99-1.10) | 0.115 | 1.04 (1.00-1.09) | 0.076 |
| Diastolic BP (by 10 mmHg) | 0.90 (0.81-1.01) | 0.068 | 0.90 (0.82-0.99) | 0.033 |
| HDL (by 5 mg/dL) | 0.98 (0.96-1.01) | 0.166 | 0.96 (0.94-0.99) | 0.002 |
| Triglycerides (by 50 mg/dL) | 1.04 (0.99-1.08) | 0.089 | 1.02 (0.99-1.06) | 0.167 |
| BMI (by 5 Kg/m^2^) | 1.11 (1.03-1.20) | 0.005 | 1.08 (1.02-1.15) | 0.007 |
| SUA (by 1 mg/dL) | 1.07 (1.03-1.12) | 0.002 | 1.04 (1.00-1.08) | 0.029 |
| Age (by 10 years) | 1.55 (1.39-1.73) | <0.001 | 1.18 (1.08-1.29) | <0.001 |
| Known duration of diabetes (by 10 years) | 1.15 (1.05-1.26) | 0.003 | 1.07 (0.98-1.16) | 0.112 |
| eGFR (by 10 mL/min/1.73 m^2^) | 0.44 (0.41-0.47) | <0.001 | 1.04 (0.98-1.10) | 0.251 |
| HbA1c (by 1%) | 1.13 (1.06-1.21) | <0.001 | 1.14 (1.08-1.21) | <0.001 |

Multivariate models.

BMI, body mass index; BP, blood pressure; eGFR, estimated glomerular filtration rate; HbA1c, glycated haemoglobin; HDL, high-density lipoprotein cholesterol; LDL, low-density lipoprotein cholesterol; SUA, serum uric acid.

Complete-case analysis performed excluding 273 patients with incomplete data (169 males and 154 females).
