# Supplementary figures and images for: Metabolic syndrome, serum uric acid and renal risk in patients with T2D
Source: PLoS One. 2017 Apr 19;12(4):e0176058. doi: 10.1371/journal.pone.0176058 (PMC5396926; doi:10.1371/journal.pone.0176058)

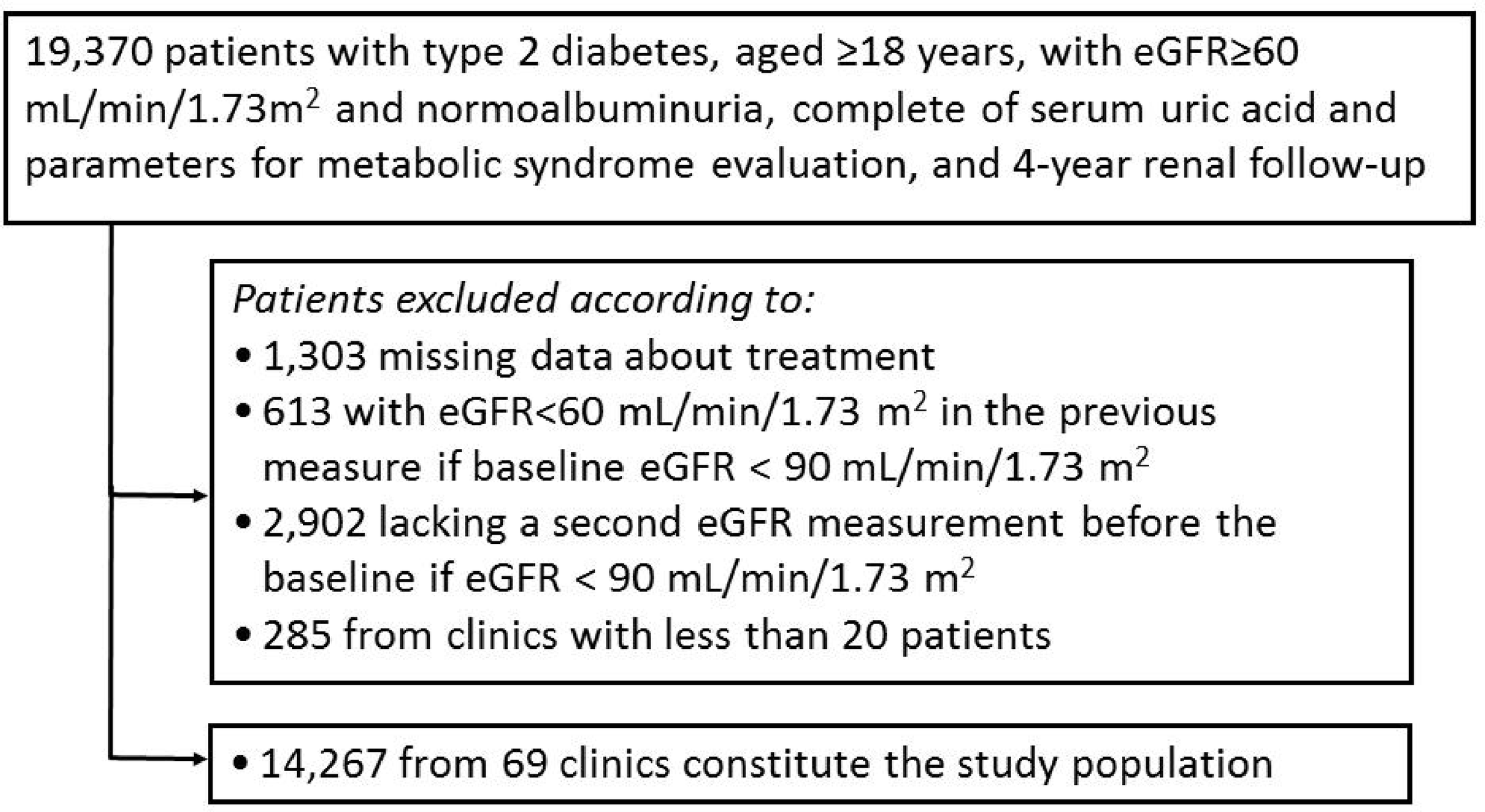

Supplement: S1 Fig — (TIFF) [file pone.0176058.s004.tiff]

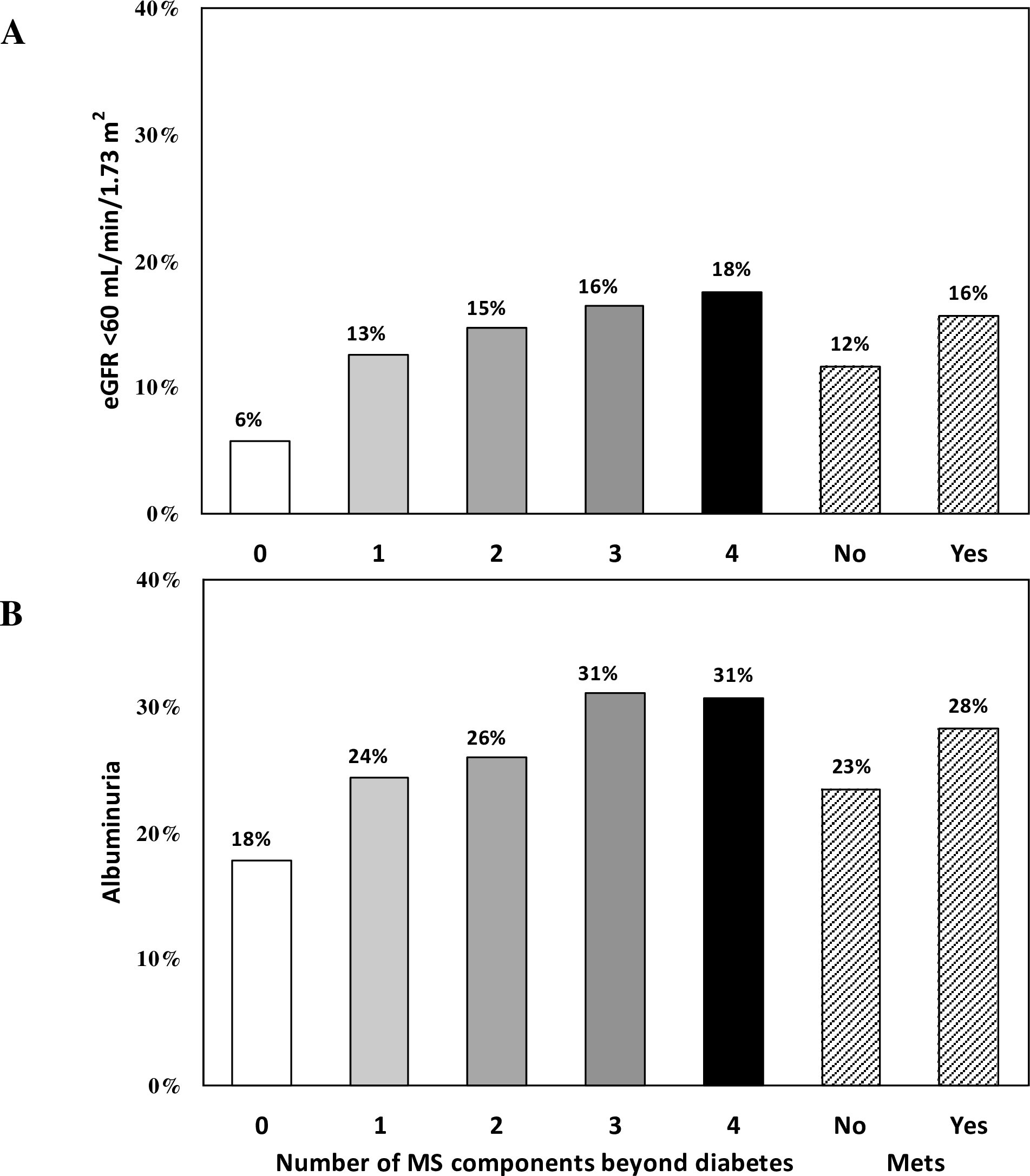

Supplement: S2 Fig — (TIFF) [file pone.0176058.s005.tiff]

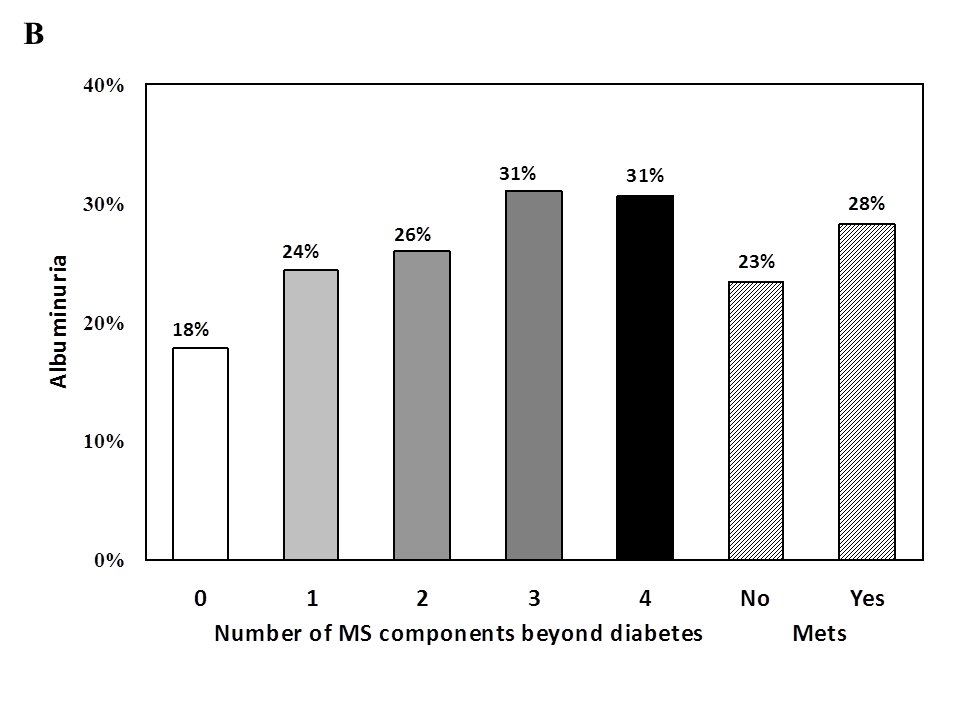

Supplement: S3 Fig — (TIF) [file pone.0176058.s006.tif]
