# Supplementary material for: Metabolic syndrome, serum uric acid and renal risk in patients with T2D
Source: PLoS One. 2017 Apr 19;12(4):e0176058. doi: 10.1371/journal.pone.0176058 (PMC5396926; doi:10.1371/journal.pone.0176058)
Supplement: S1 File — (PDF) [file pone.0176058.s007.pdf]

AMD ANNALS Study Group:

Editorial Board (in alphabetical order): Cimino Antonino[1], Fava Danila[2], Giorda Carlo Bruno[3], Meloncelli Illidio[4], Nicolucci Antonio[5], Pellegrini Fabio[5], Rossi Maria Chiara[5], Turco Salvatore[6], Vespasiani Giacomo[4]

Statistical analysis and Coordinating centre: Pellegrini F[5], Graziano G[5], Lucisano G[5], Memmo R[5], Pellicciotta E[5].

Affiliations: [1]Spedali Civili, Diabetes Unit - Brescia; [2]San Giovanni Addolorata Hospital, Diabetes and Metabolism Unit - *Roma; [3]ASL TO5, Diabets Unit - Chieri (TO); [4]Madonna del Soccorso Hospital, Diabets Unit - San Benedetto del Tronto (AP); [5]*Department of Clinical Pharmacology and Epidemiology*, Consorzio Mario Negri Sud, Santa Maria Imbaro (CH).*

Regional Tutors (in alphabetical order by region): Paciotti V, Pupillo M – Abruzzo; Armentano G, Giovannini C – Calabria; Armentano V, Laudato M, Turco S – Campania; Acquati S, Ciardullo AV, Laffi G - Emilia Romagna; Felace G, Taboga C , Tortul C - Friuli Venezia Giulia; Santantonio G, Suraci C – Lazio; Ghisoni G, Raffa M – Liguria; Genovese S, Lovagnini-Scher CA, Rampini P, Rocca A, Ruggeri P – Lombardia; Tortato E, Cotti L – Marche; Cristofaro MR, Tagliaferri M – Molise; Comoglio M, Fornengo R – Piemonte; De Cosmo S, Gentile FM - Puglia; Gigante A, Mastinu F – Sardegna; Di Benedetto A, Pata P – Sicilia; Arcangeli A, Orsini P – Toscana; Acler P, De Blasi G - Trentino Alto Adige; Cicioni G, Pocciati S – Umbria; Marangoni A, Nogara A – Veneto.

Participating centres (in alphabetical order by town): Lanero M, Bertero MG, Damassino R, Bergonzini C, Schumtz L, Seksich L - ACQUI TERME (AL); Pipitone A – ADRIA (RO); Boaretto M, Manfroi I, Parmesan L, Conte B, Soccol F – AGORDO (BL); Pagano A, Papini E, Rinaldi R,Petrucci L, Graziano F, Chianelli M, Silvagni S - ALBANO LAZIALE (RM); Rosco M – ALBEROBELLO (BA); Ansaldi E, Malvicino F, Battezzati M, Maresca P, Palenzona C – ALESSANDRIA; Boemi M, Rabini RA, Brandoni G, Lanari L, Gatti C, Testa I – ANCONA; Cherubini V – ANCONA;Doveri G, Pecorelli L, Ciccarelli A, Gallardini MB, Courthoud R, Sara Bredy S – AOSTA; Ricciardi GP – APRILIA (LT); Vitalone G, Setti D, Contrini P – ARCO (TN); Corsi A, Ghigliotti V, Oddone G, Ponzani P, Valbonesi G – ARENZANO (GE); Mazzini V – ARGENTA (FE); Di Berardino P, Colleluori P, Montani V, Trosini V – ATRI (TE); Velussi M – AURISINA (TS); Paciotti V, Alfidi P, Verdecchia B, Baliva L, Di Pietro A, Franchi G, Luce RP – AVEZZANO (AQ); Marangoni A, Pianta A, Ferrari M, Balzano S, Beltranello G - BASSANO DEL GRAPPA (VI); Dal Fabbro S, Aricò CN, Cervo L, Zanon R, Rossa S – BELLUNO; Rosco M, Di Pace MC – BISCEGLIE (BAT); Laffi G, Ciavarella A, Giangiulio S, Grimaldi M, Mustacchio A, Santacroce G - BOLOGNA S. ORSOLA MALPIGHI; Fattor B, Monauni T, Cristini M, Orion G, Crazzolara D, Amor F, Eisath JE, Lintner S – BOLZANO; Garavelli S, Calari T, Marini P, Sandri O, Scala M, Stroppa C, Trentin A - BORGO VALSUGANA (TN); Garavelli S, Calari T, Marini P, Carlin R, Carli B, Sandonà M - BORGO VALSUGANA (TN); Garavelli S, Calari T, Marini P, Zortea C, Bonet L, Pradel L, Reato S - BORGO VALSUGANA (TN); Buschini M, Bonfiglioli D, Mones D, Beldì F – BORGOMANERO (NO); Morea A, Bondesan L, Perbellini S – BOVOLONE (VR); Cimino A, Valentini U, Agosti B, Corsini R, Girelli A, Zarra E, Rocca L - BRESCIA; De Blasi G, Bergmann M, Pradi I, Unterkircher S, Piok M, Pichler M – BRESSANONE (BZ); Trinchera A, Palamà G, Palma P – BRINDISI; Carboni L, Murtas MG, Mudadu T, Turco MP, Floris M, Delogu A, Farris L – CAGLIARI; Songini M, Piras G, Seguro R, Floris R, Corona G, Lai M, Piras E – CAGLIARI; Contini PP, Cocco S, Pilosu RM, Sannia MC, Spanu F – CAGLIARI; Busciantella Ricci N, Cartechini MG, Agostinelli G, Fiorelli C – CAMERINO(MC); Nuzzi A, Ballauri C – CANALE (CN); Giorda CB, Lesina A, Romeo F – CARMAGNOLA (TO); Ciardullo AV, Giudici G, Maciejewska EG, Deroma A, Paduano M, Rossi L, Vagnini C – CARPI (MO); Dolci M, Mori M, Baccetti F, Gregori G – CARRARA (MS); Straface E – CASALBORDINO (CH); Pozzuoli G, Laudato M, Barone M, Stasio GB – CASERTA; Tondini S, Borgoni F - CASTEL DEL PIANO (GR); Grosso J, Rossi L, Scarsellato C, Sciulli A, De Marco F - CASTEL DI SANGRO (AQ); Confortin L, Marin N, Lamonica M – CASTELFRANCO (TV); Gialdino S – CASTROVILLARI (CS); Borzì V, Gatta C, Rapisard R, Strano S, Calabrò M – CATANIA; Puccio L – CATANZARO; Zolli M, Coracina A – CAVARZERE (VE); Starnone V, Del Buono A, Terracciano AM – CELLOLE (CE); Monda MV – CENTO (FE); Castro F, Guaglianone A, Maccari V – CETRARO (CS); Corsi L, Versari G, Falivene MR, Boletto N, Corsi S – CHIAVARI (GE); Giorda CB, Marafetti L – CHIERI (TO); Vitacolonna E, Capani F, Caputo L, Di Nisio L, Simonetti F – CHIETI; Boscolo Bariga A, Nogara A, Ballarin G, De Boni S, Di Benedetto S – CHIOGGIA (VE); Chiambretti AM, Fornengo R, Di Vito L, Pascuzzo MD, Urli P – CHIVASSO (TO); Rocca A, Rumi P, Balzarini B, Galli P, Castellan M, Giannetti A, Russotti C, De Blasi A, Perna A - CINISELLO BALSAMO (MI); Campanelli C, Ranchelli A, Biccheri D, Dadi G - CITTA' DI CASTELLO (PG); Santantonio G, Massa L, Baldi GP, Sciacca F, Costanzo E, Spada M, Paolini G – CIVITAVECCHIA (RM); Ziller P, Portolan F, Pasolini G – CLES (TN); Ghilardi G, Fiorina P – CLUSONE (BG); Grata ML – CODIGORO (FE); Capretti L, Speroni G, Fugazza L – CODOGNO (LO); Massafra C, Lovagnini Scher A - COLOGNO MONZESE (MI); Cimicchi MC, Percudani C, Risolo T, Saccò P – COLORNO (PR); Grata ML –

COMACCHIO (FE); Gidoni Guarnieri GL, Piccolo D, Bravin C, De Noni E, Scarpel M, Marcon M, Giacon F – CONEGLIANO (TV); Panebianco G, Tadiotto F, Da Tos V, D'Ambrosio M – CONSELVE (PD); Pellizzola D, Zampini MA, Frezzati E, Mari E, Raminelli E – COPPARO (FE); Gaiti D, Bosi EA, Chierici G, Pilla S, Copelli M, Zanichelli P, Bertelli L, Caretta P, Vezzani V, Bodecchi S – CORREGGIO (RE); Longobucco A – COSENZA; Ruggeri P, Di Lembo S, Spotti E, Carrai E, Degli Innocenti A, Manini L, Persico R, Rossi C – CREMONA; Magro G – CUNEO; Marelli G, Vilei V, Andrioli M, Bellato L, Fedeli M, Merlini A, Pinelli G – DESIO (MI); Marin G, Contin ML, Gallo A, Parlato P, Pecchielan W, Jacovacci J – DOLO (VE); Placentino G – DOMODOSSOLA (VB); Richini D, Molinari S, Strazzeri R – ESINE (BS); Panebianco G, Tadiotto F, Da Tos V, D'Ambrosio M – ESTE (PD); Fabbri T, Di Bartolo P – FAENZA (RA); Cotti L, Garrapa G – FANO (PU); D'Incau F, Lagomanzini P, Conte P, Todesco F – FELTRE (BL); Foglini P, Tortato E, Pantanetti P, Bedetta C, Maricotti R – FERMO; Tomasi F, Monesi M, Graziani R, Beretta F, Penna L – FERRARA; Guberti A, Dazzi D – FIDENZA (PR); Dolci M, Mori M, Baccetti F, Gregori G – FIVIZZANO (MS); Pocciati S – FOLIGNO (PG); Forte E, Gasbarrone A, Marrocco T, Moschetta R – FONDI (LT); Tuccinardi F, De Meo F, Forte E, Coppola A, Pirolozzi P, Placitelli E, Vallefuoco R – GAETA (LT); Taboga C, Catone B, Ceschia S, Urban M - GEMONA DEL FRIULI (UD); Ghisoni G, Fabbri F, Torresani M, Crovetto R – GENOVA; Corsi A, Battistini M, Fabbri F, Carosia P – GENOVA; Viviani GL, Durante A, Pais F, Lilliu V – GENOVA; Rosco M, Quieto C - GIOIA DEL COLLE (BA); D'Ugo E, Squadrone M, Amenduni T, Iovannisci MM, Della Penna L, Potente F, Delle Donne T, Massa C, Ulisse MA – Gissi (CH); De Berardinis S, Guarnieri I, Pace S, Splendiani M, Di Giuseppe R – GIULIANOVA (TE); Tortul C, Brunato B, Assaloni R, Muraro R, Loro R, Bucciol S – GORIZIA; Rosco M, Lavacca C – GRAVINA (BA); Rossi M, Sabbatini G, Quadri F, Sambuco L, Santacroce C – GROSSETO; Bosi EA, Chierici G, Pilla S, Gaiti, Copelli, Zanichelli, Bertelli, Paola Caretta D, Vezzani V, Bodecchi S – GUASTALLA (RE); Marino C, Micheletti A, Petrelli A – GUBBIO (PG); Corda A, Pisano L, Guaita G, Deias C – IGLESIAS (CI); Trevisan G, Coletti I – JESOLO (VE); Iannarelli R - L'AQUILA; Pupillo M, De Luca A, Minnucci A, Antenucci D, Di Florio C, Angelicola G, Bosco A, Fresco R, Di Marco G – LANCIANO (CH); Ugolotti D, Cadossi T, Ferrari M – LANGHIRANO (PR); Tagliaferri M, Di Caro P, Mazzocchetti M – LARINO (CB); Buzzetti R, Leto G, Gnessi C, Cipolloni L, Foffi C, Moretti C, Venditti C – LATINA; Morea A, Bondesan L, Perbellini S – LEGNAGO (VR); Meniconi R, Bertoli S, Cosimi S - LIDO DI CAMAIORE (LU); Di Cianni G, Orsini P, Turco A, Richini A, Marconi S, Sannino C, Lemmi P, Giuntoli S, Manfrè N – LIVORNO; Giannini F, di Carlo A, Casadidio I – LUCCA; Melandri P, Di Bartolo P – LUGO (RA); Maolo G, Polenta B, Piccinini N – MACERATA; Pozzuoli G, Laudato M, Barone M, Stasio GB – MADDALONI (CE); Vincenti C, Pastore N, Mega P, Magurano E, Cananiello A – MAGLIE (LE); Francescutto CA, Brussa Toi E, Gaspardo G, Angeli L, Ronchese L – MANIAGO (PN); Sciangula L, Ciucci A, Contartese A, Banfi E, Castelli E - MARIANO COMENSE (CO); Tatti P, Bloise D, Di Mauro P, Masselli L – MARINO (RM); Lo Presti A, Scarpitta AM, Gambina F – MARSALA (TP); Venezia A, Morea R, Lagonigro G, Copeta G, Iannucci V, Milano V, Trupo M – MATERA; Lochmann A, Marchetto PE, Incelli G, De Paola G, Steiger MM, Gamper MA, Breitenberger S, Holzner M, Frischmann J – MERANO (BZ); Lambiase C, Di Vece T , D'Aniello M, Fezza M, Giordano C, Leo F - MERCATO S. SEVERINO (SA);Saitta G – MESSINA; Di Benedetto A, Cucinotta D, Di Vieste G, Pintaudi B – MESSINA; Pata P, Mancuso T – MESSINA; Musacchio N, Giancaterini A, Lovagnini Scher A, Pessina L, Salis G, Schivalocchi F – MILANO; Testori G, Rampini PA, Cerutti N, Morpugo PS, Cavaletto ML, Bonino G, Morreale F – MILANO; Mariani G, Ragonesi PD, Bollati P, Colapinto P – MILANO; Bosi E, Falqui L – MILANO; Bortolato L, Cosma A, Pistolato P, Centenaro B, Ceccato A; MIRANO (VE); Campobasso G – MODUGNO (BA); Gentile FM, Zaurino F, Mazzotta G - MOLA DI BARI (BA); Comoglio M, Manti R, Giorda CB – MONCALIERI (TO); Tortul C, Da Ros R, Carlucci S, Narduzzi L, Bortolotto D, D'Acunto L, Stanic L, Brunato B, Assaloni R – MONFALCONE (GO); Volpi A, Coracina A, Cospite AM – MONTEBELLUNA (TV); Manicardi V, Michelini M, Finardi L, Borghi F, Manicardi E - MONTECCHIO EMILIA (RE); Lombardi S, Tommasi C, Iaccarino M, Cozza S, Binotto M, Marini F, Mecenero I, Massignani S, Stecco P, Urbani E, Massariol W, Parolin R - MONTECCHIO MAGGIORE (VI); Gatti A, Bonavita M, Creso E, Giannettino R, Gobbo M – NAPOLI; Turco S, Iovine C, Turco AA, Riccardi G – NAPOLI; Iazzetta N, Giannattasio C – NAPOLI; Armentano V, Egione O, Galdieri S, Velotti A, Azzolina A, Annicelli G – NAPOLI; Sorrentino T, Gaeta I, Del Buono A – NAPOLI; Zenari L, Bertolini L, Sorgato C, Grippaldi F – NEGRAR (VR); Stroppiana M, Popolizio R, Carbone N, Grasso S, Abate S, Gaggero GC - NIZZA MONFERRATO (AT); Strazzabosco M, Brun E - NOVENTA VICENTINA (VI); Carlesi GP, Garrone S - NOVI LIGURE (AL); Gigante A, Cicalò AM, Clausi C, Cau R – NUORO; Manconi A, Carboni A, Angius MF, Pinna AA, Caria S, Filigheddu GD, Tonolo G, Carta I – OLBIA (OT); Calebich S, Burlotti C – OME (BS); Saglietti G, Placentino G, Schellino A – OMEGNA (VB);Mastinu F, Madau G, Cossu M, Mulas F, Zoccheddu S – ORISTANO; Balsanelli M, Fetonti M, Rotolo A, Sambo P – OSTIA (RM); Secchi E, Angotzi MA, Loddoni S, Brundu I, Careddu F, Becciu A, Gabriella Piras G – OZIERI (SS); Novara F, Cipro F – PACECO (TP); Torchio G, Palumbo P, Bianchi A, Colucci G, La Motta G - PADERNO DUGNANO (MI); Tiengo A, Avogaro A, Bruttomesso D, Crepaldi C, Fadini G, Guarnieri G, Lavagnini MT, Maran A, Vedovato M, de Kreutzenberg V – PADOVA; Fedele D, Lapolla A, Sartore G, Bax G, Cardone C, Dalfrà MG, Masin M, Toniato R, Francesco Piarulli – PADOVA; Mattina G – PALERMO; Fulantelli MA – PALERMO; Gioia D, Conti M – PALERMO; Ridola G – PALERMO; D'Agati F – PALERMO; Grossi G, De Berardinis F – PAOLA (CS); Zavaroni I, Dei Cas A, Franzini L, Usberti E, Antonimi M, Anelli N, Poli R, Ridolfi V, Michela M, Haddoub S, Prampolini G, Muoio A – PARMA; Cimicchi MC, Ugolotti D, Filippi D, Ferrari M, Bucci F – PARMA; Tardio SM, Calderini MC, Magotti MG, Quarantelli C, Vernazza MA, Carolfi A, Saracca R – PARMA; Picchio E, Del Sindaco P – PERUGIA; Spalluto A, Maggiulli L, Torreggiani V, Rastelletti S, Ugolini C,

Pucci N, Magi S, Muratori S – PESARO; La Penna G, Consoli A – PESCARA; Galeone F, Magiar AV – PESCIA (PT); Gherardini V, Moretti L, Bientinesi M, Landi L, Bernardi A – PIOMBINO (LI); Del Prato S, Miccoli R, Bianchi C, Penno G, Venditti F – PISA; Anichini R, De Bellis A, Bruschi T, Butelli L, Gioffredi M, Gori R, Picciafuochi R, Malagoli R, Bernini A - PISTOIA; Gelisio R, Zanon M, Del Bianco A, Bamiston A, Signorato M – PORTOGRUARO (RO); Mazzini V – PORTOMAGGIORE (FE); Citro G – POTENZA; Arcangeli A, Calabrese M, Ianni L, Lorenzetti M, Marsocci A, Guizzotti S, Memoli G – PRATO; Cabasino F, Farci F, Atzori A, Sanna A, Ghiani M, Siotto I, Sedda M, Manis A, Loddo C, Loddo I, Pisano L, Seguro P, Cuomo A, Orlando L, Olanda GB - QUARTU SANT'ELENA (CA); Pucci A - QUATTROMIGLIA DI RENDE (CS); Massenzo M - QUATTROMIGLIA DI RENDE (CS); Di Bartolo P, Sardu C – RAVENNA; Giovannini C - REGGIO CALABRIA; Perrone G, Corazziere F, La Puzza I - REGGIO CALABRIA; Tripodi PF, Riggio S, Giampaolo A - REGGIO CALABRIA; Mannino D - REGGIO CALABRIA; Aleandri AR, Guidi MV, Battisti B, Faraglia MR, Lilli V – RIETI; Leotta S, Suraci C, Visalli N, Gagliardi A, Fontana L, Altomare M, Carletti S, Abbruzzese S – ROMA; Chiaramonte F, Giordano R, Rossini M, Migneco G – ROMA; Cappelloni D, Urbani A – ROMA; Piergiovanni F, Fava D, Simonetta A, Massimiani F – ROMA; Bulzomì R – ROMA; Giuliano M, Pennafina MG, Di Perna P – ROMA; D'Accinni MP, Paolucci D, D'Ubaldi A, D'Angelo MT, Masaro G, Pietrantoni M, Fratini M, La Rosa R – ROMA; Poggi M, Piccirilli F, Pisano R, Saponara C, Conforti I, Penza A - ROMA; Scalpone R, Lo Pinto S, Iacovella L, Caccamo C, Sposito S, Teodonio C - ROMA; Armentano G, Restuccia MG, Mirto G – ROSSANO (CS); Girardello R, Gennaro R, De Moliner L, Bettini E, Mattuzzi A, Speese K, Frisinghelli F – ROVERETO (TN); Genovese S, Locatelli F – Rozzano (MI); Nicoletti M, Trojan N, Centis R - S.VITO AL TAGLIAMENTO (PN); Li Volsi P, Levis E, Zanette G – SACILE (PN); Comba G, Ballatore L – SALUZZO (CN); Cattaneo A, Aglialoro A, Guido R, Patrone M, Zecchini M – SAMPIERDARENA (GE); Vespasiani G, Meloncelli I, Clementi L, Galetta M, Marconi V - SAN BENEDETTO DEL TRONTO (AP); Bordin P, Perale L - SAN DANIELE DEL FRIULI (UD); Vinci C, Sira Zanon M, Geretto L, Toffolo C, Furlan MG, Mazzanti G - SAN DONÀ DI PIAVE (VE); Vinci M, Gelisio R - SAN DONA' DI PIAVE (VE); Sica V, Armeni M, Derai R, Ennas O, Mamusa S, Pisano MA, Carreras L - SAN GAVINO MONREALE (SV); De Cosmo S, Rauseo A - SAN GIOVANNI ROTONDO (FG); Cervone S, Leggieri A, Pontonio M - SAN MARCO IN LAMIS (FG); Sturaro R, Raffa M, Quattrocchi F, Molinaro M, Trasatti M, Ferretti B – SANREMO (IM); Rosco M, Labarile G – SANTERAMO (BA); Baule GM, Gentilini A, Spanu MA, Fancellu A, Bianco P – SASSARI; Lione L, Massazza G, Bocchio G, Bosco E – SAVONA; Monachesi M, Carta G, Boschetti M, Ceresola E, Venier E – SAVONA; Calcaterra F, Cataldi F, Miola M – SCHIO(VI); Manfrini S – SENIGALLIA(AN); Lai A, Locci B, Putzu D – SENORBI (CA); Tanganelli I, Leonini M – SIENA; Egger K, Marchiotto W – SILANDRO (BZ); Vincis L, Orlandini V, Pilloni C, Farci R, Pelligra I, Renier G - SIRAI – CARBONIA; Mameli M, Pala A, Devigus E – SORGONO (NU); Felace G, Fumagalli I – SPILIMBERGO (PN); Lalli C, Leandri M, Agliani M, De Pascalis L – SPOLETO (PG); Malci F, De Ciocchis A – SUBIACO (RM); Diodati MB, Macerola B – SULMONA (AQ); Davì S, Caccavale A, Brocato L, Pognant Gros M, Borla S - SUSA (TO); Lattanzi E, Piersanti C, Piersanti A, Spinelli I, Tuzzoli L, Tulini V, Quaranta G, Iorio V, Tirabovi M – TERAMO; De Candia - TERLIZZI (BA); Cicioni G, Massarelli MG, Venturi S – TERNI; Travaglini A, Draghi P – TERNI; Pomante P - TOCCO DA CASAURIA (PE); Richiardi L, Clerico A – TORINO; Bruno A, Cavallo Perin P, Ghigo E, Porta M, Scuntero P, Arcari R, Bertaina S, Bo S, Broglio F, Bruno G, Degiovanni M, Fornengo P, Grassi G, Inglese V, Maccario M, Maghenzani G, Marena S, Martina V, Passera P, Ruiu G, Tagliabue M, Zanone M – TORINO; Monge M, Boffano GM, Macrì K, Maio P – TORINO; Ozzello A, Pergolizzi E, Gaia D, Gennari P, Micali G, Rossetto E, Dalmazzo C, Oreglia M, Stefani T – TORINO; Dossena C, Paglia P, Bosoni S – TORTONA (AL); Acler P, Romanelli T, Inchiostro S, Dauriz M – TRENTO; Bossi CA, Meregalli G, Balini A, Berzi D, Filippini B, Crotto G – TREVIGLIO (BG); Paccagnella A, Orrasch M, Sambataro M, Citro T, Kiwanuka E, Bagolin E, Almoto B – TREVISO; Macchia A, Branca MT, Filesi M – TRICASE (LE); Candido R, Caroli E, Manca E, Petrucco A, Tommasi E, Jagodnik G, Baskar B, Daris N, Dal Col P – TRIESTE; Pellegrini MA, Tonutti L, Venturini G – UDINE; Andreani M, Turchi F, Fedrighelli F, Martinelli G – URBINO; Sposito S, Rongioletti R, Candidi M – VELLETRI (RM); Pais M, Moro E – VENEZIA; Cervellino F, Sinisi R, Zampino A – VENOSA (PZ); Saglietti G, Placentino G, Schellino A - VERBANIA PALLANZA (VB); Mingardi R, Lora L, Reitano R, Stocchiero C – VICENZA; Strazzabosco M, Brun E, Simoncini M, Mesturino CA, Zen F – VICENZA; Di Pietro S, Scoponi C, Tilaro L, Pelliccioni S, Slongo R, Vita E; VITERBO; Garofalo A, Vitale F, Campanella B – VITTORIA (RG); Mastrilli V, Del Buono A, Borrelli T, D'Avino A – VOLLA (NA); Morea A, Perbellini A, Bondesan L – ZEVIO (VR).
